# Suppression of Autoimmune Retinal Inflammation by an Antiangiogenic Drug

**DOI:** 10.1371/journal.pone.0066219

**Published:** 2013-06-13

**Authors:** Takeru Yoshimura, Ofra Benny, Lauren Bazinet, Robert J. D’Amato

**Affiliations:** 1 Department of Surgery, Vascular Biology Program, Boston Children’s Hospital, Harvard Medical School, Boston, Massachusetts, United States of America; 2 Department of Ophthalmology, Boston Children’s Hospital, Harvard Medical School, Boston, Massachusetts, United States of America; New York University, United States of America

## Abstract

Chronic and recurrent uveitis account for approximately 10% of legal blindness in the western world. Autoimmune uveitis is driven by activated CD4^+^ T cells that differentiate into effector T helper cells (Th1, Th2, and Th17) which release proinflammatory cytokines that damage the retina. In this study we investigated the effect of the methionine aminopeptidase 2 (MetAP2) inhibitor, Lodamin, on T cell activation and differentiation. MetAp2 is an enzyme which regulates cellular protein synthesis and is highly expressed in T cells. Lodamin was found to suppress T cell receptor (TCR) mediated T cell proliferation and reduced the production of Th1 and Th17 cells. Further, Lodamin suppressed overall inflammation in the mouse model of experimental autoimmune uveitis (EAU) by a six fold. This effect was attributed in part to a reduction in retinal proinflammatory cytokines, down regulation of MetAP2 expression in purified lymph node CD4^+^ T cells, and a general normalization of the systemic immune reaction.

## Introduction

Autoimmune uveitis is a complex, sight-threatening condition associated with a multitude of diseases. It affects 2 million Americans, accounting for about 10% of the severe visual impairments in the United States [1]. This condition presents either as isolated intraocular inflammation or as part of systemic autoimmune diseases such as Behcet’s disease, sarcoidosis, Vogt-Koyanagi-Harada (VKH) disease, or ankylosing spondylitis. Posterior uveitis is characterized by inflammation in the vitreous, retina, and choroid, with associated vision loss due to damage of the photoreceptor cells [2]. The specific mechanism by which the pathological process is triggered is often unknown, but, as is the case in other autoimmune inflammatory diseases, T cells have been shown to play a central role. The transformative nature and plasticity of T cell differentiation is an important process in the progression of autoimmune disease. Naive CD4^+^ T cells activate and differentiate into Th1 cells upon interleukin 12 (IL-12) stimulation or Th2 upon interleukin-4 (IL-4) stimulation. These cell populations contribute to the cellular immune reaction locally in the eye, as well as activate the humoral immune response in a systemic autoimmune reaction.

The recent identification of a highly proinflammatory subpopulation of T cells, the Th17 effector cell subset, has focused attention on its role in the pathogenesis of autoimmune disease. These cells produce the proinflammatory cytokine interleukin-17 (IL-17) which can recruit monocytes to an inflammation site, induce neutrophil mobilization, and trigger the cascade-like release of additional cytokines [3]. T cells can differentiate into IL-17-producing effector cells when stimulated by Interleukin 6 (IL-6), transforming growth factor β (TGF-β), and Interleukin 23 (IL-23). Alternatively, Th17 cells can differentiate into Th1 producing interferon-gamma (IFN-γ) through an IL-12 trigger.

Recent publications have shown autoantigen-specific Th17 cells to be the pathogenic T cell subset in both the endogenous autoimmune uveitis model (EAU, the murine model of autoimmune uveitis) [4] and the experimental autoimmune encephalomyelitis model (EAE, the murine models of multiple sclerosis) [3]. In humans, Th17 cells have been identified in the central nervous system of patients with multiple sclerosis [3]. Additionally, IL-17A has been found to be increased in patients with uveitis [4], [5]. These findings suggest that Th17 and IL-17 may be therapeutic targets in autoimmune diseases.

Current treatments for uveitis are mainly based on broad spectrum immunosuppressants like corticosteroids that suppress acute inflammation, or other agents such as cyclosporin A and methotrexate that decrease chronic ocular inflammation. However, immunosuppressive treatments target multiple cells, such T cells, B cells, and cells of the innate immune system. Their long-term systemic administration can cause severe side-effects including development of glaucoma, osteoporosis, infertility, liver and kidney dysfunction, and secondary malignancy [6]. A new generation of more specific protein-based drugs, such as Infliximab, an antibody against TNF-α, and tocilizumab, a recombinant humanized anti-IL-6 receptor antibody [7], [8], have been reported as effective in multiple autoimmune inflammatory diseases. These agents are active in uveitis with reduced immunosuppressive side-effects. However, these drugs have their own systemic toxicities such as neurological and cardiac complications and adverse coetaneous reactions [9]. Due to these toxicities, there is a great need to search for novel targets in uveitis and other autoimmune diseases which will offer safer therapeutic approaches with comparable or improved efficacy.

In this study we investigate a potential therapy which has a mechanism of action distinct from the existing therapies. Lodamin, an oral polymeric formulation of TNP-470, is an irreversible inhibitor of MetAP2 [10]. MetAP2 has been recognized as a key target in angiogenesis [11]. This enzyme is over expressed in proliferating endothelial cells and is involved in protein synthesis during endothelial cell proliferation. Inhibition of MetAP2 was found to cause cell cycle arrest through p53 activation and induction of the cyclin-dependent kinase inhibitor p21(CIP/WAF) [12],[13]. Aside from endothelial cells, T cells express high levels of MetAP2 (Figure S1). Here we demonstrate that Lodamin suppresses T cell activation, migration and differentiation into Th1/Th17 cells. Moreover, we show the ability of Lodamin to inhibit uveitis in the EAU model.

## Results

### Lodamin has Rapid Internalization Kinetics into T Cells

**Figure 1:**
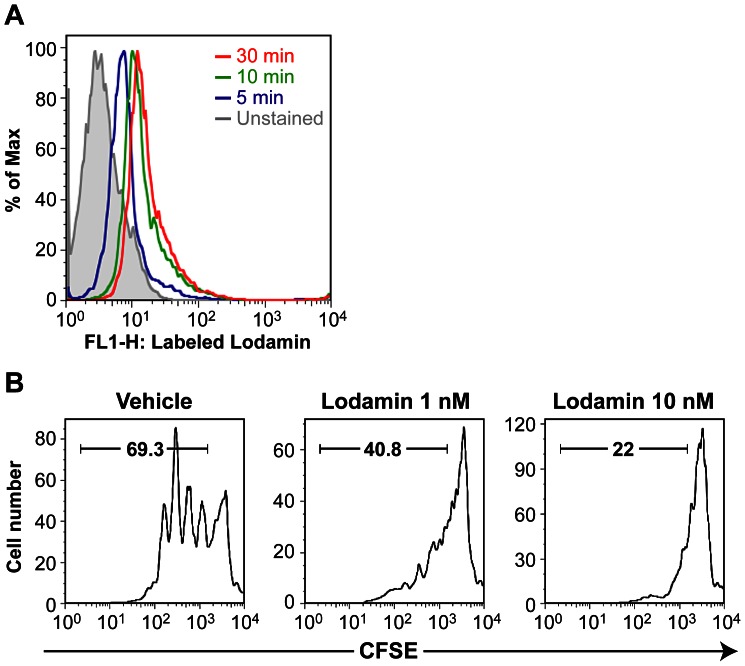
Lodamin internalizes into T cells and suppresses anti-CD3 mediated T cell proliferation *in vitro.* (A) Uptake of 6-coumarin-labeled Lodamin by anti-CD3 stimulated T cell as detected by FL1 channel in flow cytometry. (B) To assess cell proliferation, splenocytes from C57BL/6J mice were labeled with 10 nM CFSE then stimulated with 1 µg/ml of soluble anti-CD3 antibody for 72 h. CFSE fluorescence intensity was analyzed by flow cytometry. Data are representative of three independent results.

To determine the uptake of Lodamin by T cells (CD3 positive cells), we used fluorescently labeled Lodamin. Lodamin exhibited rapid internalization kinetics into anti-CD3 stimulated T cells indicated by the shift of mean fluorescent signal peak over time in flow cytometry (FACS). As shown in Figure 1A, 6-coumarin fluorescent signal (FL-1^high^) was detected in T cells as early as 5 minutes post incubation, and enhanced over time (at 10 min and 30 min), indicating further cellular internalization. These results demonstrate the endocytosis of Lodamin into activated T cells.

### Lodamin Inhibits anti-CD3-mediated T Cell Proliferation *in vitro*

To investigate the effect of Lodamin on anti-CD3-mediated T cell proliferation, whole splenocytes were stained with carboxyfluoroscein succinimidyl ester (CFSE) and stimulated with soluble anti-CD3 antibody. 72 hours after cell stimulation, splenocytes were stained with anti-CD4 antibody and proliferation of CD4^+^ cells was assessed by CFSE-dilution using flow cytometry. Figure 1B shows that, compared to control, a single dose of Lodamin inhibited cell proliferation, as shown by a higher number of CFSE stained cells in stimulated populations of CD4^+^ cells (marker indicates CFSE dilution: 40.8% at 1 nM, 22% at 10 nM, respectively, compared with 69.3% of control).

### Lodamin Inhibits Anti-CD3-mediated T Cell Proliferation and Th1/Th17 Cell Differentiation *in vitro*

**Figure 2:**
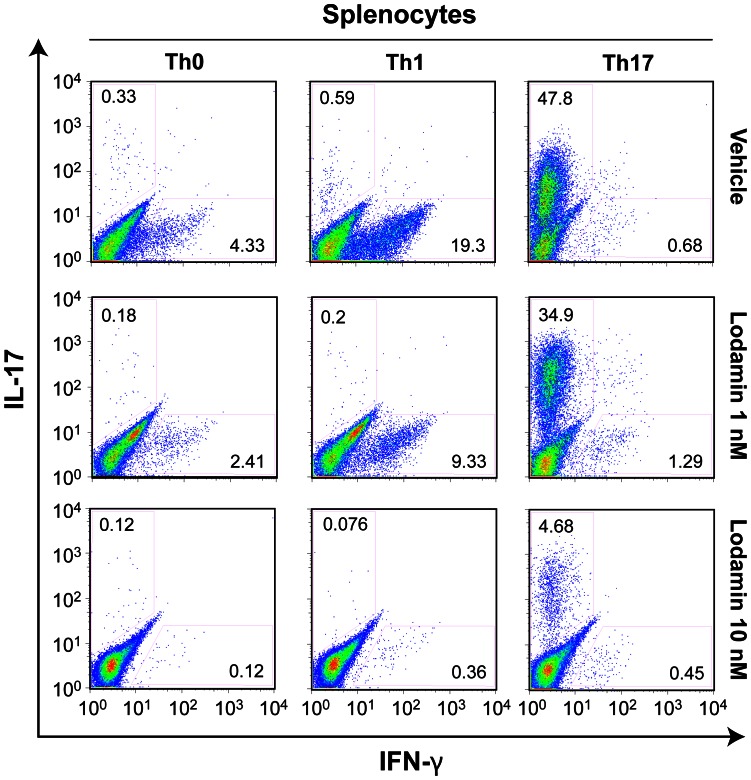
Lodamin inhibits Th1/Th17 cell differentiation. Whole splenocytes were activated by anti-CD3 antibody for 3 days under Th1 or Th17 polarization conditions in the presence or absence of Lodamin (vehicle, 1 nM and 10 nM), followed by stimulation with PMA and ionomycin in the presence of Brefeldin A; Cell were analyzed by FACS following intracellular cytokine staining of IL-17 and IFN-γ, all plots are gated on CD4+ cells. Data are representative of three independent results.

**Figure 3:**
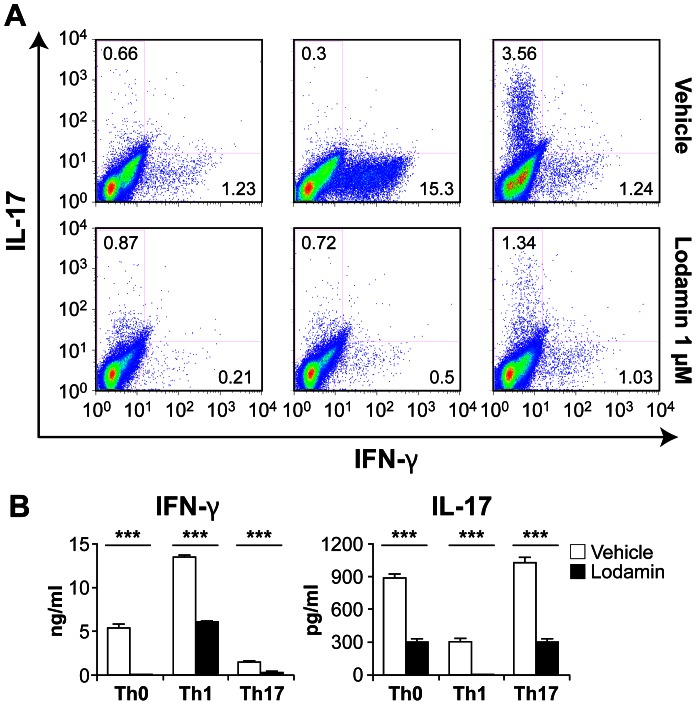
Lodamin inhibits Th1/Th17 cell differentiation by inhibiting cell proliferation and reducing the production of proinflammatory cytokines. (A) Purified CD4+CD25- T cells from spleen were activated by anti-CD3 antibody for 3 days under Th1 or Th17 polarization conditions in the presence or absence of Lodamin (1 nM), followed by stimulation with PMA and ionomycin in the presence of Brefeldin A; all plots are gated on CD4+ cells. Polarization conditions: Th0; anti-IFN-g Ab (10ug/ml) and anti-IL-4 Ab (10ug/ml); Th1; IL-12 (10 ng/ml) and anti-IL-4 Ab (10 µg/ml); Th17; IL-6 (20 ng/ml), TGFβ (2 ng/ml), anti-IFNγ Ab (10 µg/ml) and anti-IL-4 Ab (10 µg/ml) (B) Lodamin inhibits hallmark cytokine of Th1/Th17 under each polarization condition. IFN-γ and IL-17 protein level as measured by ELISA from activated cells supernatants (as describe in panel A). (mean ± SEM, *n = 3*, ***p<0.001).

Splenocytes or purified CD4+ T cells were cultured under either Th1 promoting conditions [IL-12 (10 ng/ml) and anti-IL-4 Ab (10 µg/ml)], or Th17 conditions [IL-6 (20 ng/ml), TGFβ (2 ng/ml), anti-IFNγ Ab (10 µg/ml) and anti-IL-4 Ab (10 µg/ml)]. Splenocytes stimulated by soluble anti-CD3 Ab under Th1 or Th17 polarizing conditions were assessed for their IFN-γ and IL-17 levels using intracellular cytokine staining. Figure 2 shows a representative FACS analysis of cultured splenocytes treated with Lodamin. After 72 hours in culture, Lodamin (1 nM or 10 nM) inhibited cellular production of IL-17 and IFN-γ. Similarly, when CD4^+^CD62L^+^ naive T cells purified from the spleen were stimulated with plate-coated anti-CD3 Ab under Th1- and Th17-polarizing conditions, cultured T cells containing 1 nM Lodamin reduced IFN-γ and IL-17 production as indicated by FACS (Figure 3A) and showed significant IFN-γ and IL-17 protein reduction by ELISA (p<0.001) (Figure 3B).

### Oral Administration of Lodamin Inhibits EAU with Reduced Antigen-specific IFN-γ and IL-17 Production

**Figure 4:**
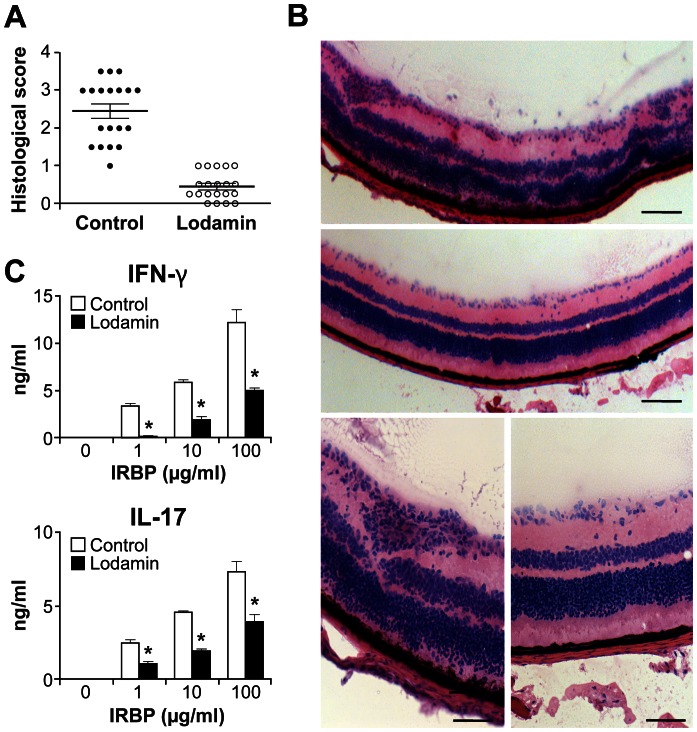
Oral administration of Lodamin reduces the severity of EAU with reduced antigen-specific Th1/Th17 reaction. (A) histological score of EAU evaluated on day 21 post immunization with IRBP_1–20_- in response for oral Lodamin treatment (30 mg/kg q.o.d) (○) or vehicle (•) (*n = 20*, mean ± SEM, *p<0.05 Mann-Whitney U test). (B) represented histopathological images of eyes enucleated from vehicle (control) or Lodamin-treated mice on day 21 post IRBP_1–20_ immunization. The control mice exhibited uveitis with focal loss of the photoreceptor layer in the choroid as well as massive infiltrates of monocytes in the retina, vitreous, and choroid with retinal folding and focal granulomatous lesions (arrows). In contrast, in Lodamin treated group only a few inflammatory cells are observed. Scale bars = 100 µm. (C) FACS analysis of IFN-γ and IL-17 production by T cells from draining lymph node. CD4^+^ T cells from Lodamin-treated mice produced significantly less IRBP-specific IL-17 as well as IFN-γ than cells from untreated mice on day 21 post after IRBP_1–20_ immunization (mean ± SEM, *n = 3*, *p<0.05).

To evaluate the effect of Lodamin on EAU, WT C57BL/6J mice were immunized with interphotoreceptor retinal-binding protein (IRBP_1–20_) as described in the Materials and Methods section and treated with Lodamin (30 mg/kg) administered orally every other day. Evaluation of EAU progression was performed 21 days post-immunization. Clinical fundus examination revealed that the severity of uveitis was ameliorated in Lodamin-treated mice compared with vehicle-treated (Figure 4A). Histopathological examination of the eyes from the control group revealed that the retinal architecture was disorganized, showing extensive retinal folding, with inflammatory cell infiltration and severe photoreceptor damage (Figure 4B). In contrast, histological examination in Lodamin-treated mice showed decreased inflammatory cell infiltration and/or granuloma formation. Histological evaluation indicated a score of 2.4±0.55 in the control group vs 0.4±0.42 with Lodamin p = 0.0109 (Figure 4A) (see Methods for reference of scoring method). The level of IFN-γ and IL-17 protein produced by T cells from a draining lymph node was examined on day 21. CD4^+^ T cells from Lodamin-treated mice showed significantly less IRBP-specific IL-17 and IFN-γ than those from untreated mice (Figure 4C) suggesting an IRBP-specific response in suppression of retinal inflammation.

### Lymph Node Size and Cellular Characterization Following Treatment

**Figure 5:**
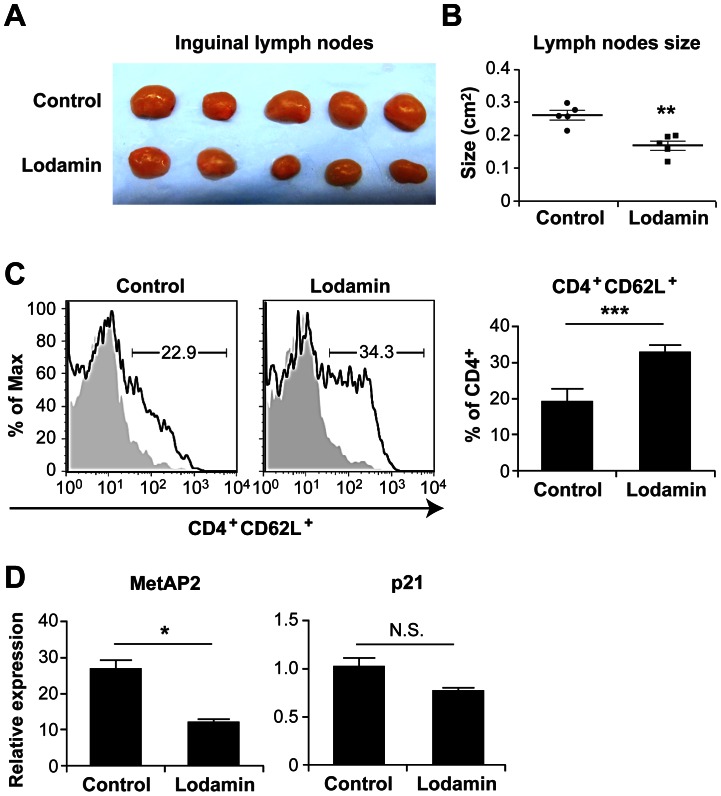
Reduced size of lymph nodes in Lodamin administered mice. On day 21 post IRBP_1–20_ immunization, lymph nodes from control vehicle-administered mice and Lodamin treated mice were collected. (A) images of lymph nodes removed at day 21. (B) volume of inguinal lymph nodes calculated with two parameters; (width)^2^×(length)×0.52. (C) diagrams (left) and statistics (right, mean ± SEM, *n = 5,* ***p<0.001)) of FACS analysis of cell surface CD4^+^CD62L^+^ expression of single cell suspension from lymph nodes. (D) MetAP2 expression and p21 expression as evaluated by qPCR from purified lymph nodes CD4^+^ T cells, (*n = 5,* *p<0.05) values represent means ± SEM.

Enlarged lymph nodes are a pathological feature of systemic inflammation. Lymph nodes of Lodamin treated EAU mice were smaller than those of control EAU-afflicted mice (Figure 5A, B). No changes in body weight or activity level were seen with treatment. To analyze T cell profile in the lymph nodes with or without treatment, we measured expression of CD62L (L-selectin) on lymphatic CD4+ cells. CD62L is a cell adhesion molecule and a “homing receptor” for T cells to enter secondary lymphoid tissues. It is highly expressed on naive T cells, and down regulated upon immune activation. The percentage of CD4^+^CD62L^+^ from each individual mouse was assessed by flow cytometry. As shown in Figure 5C, CD4^+^CD62L^+^ (L-selectin) expression from draining lymph nodes was significantly higher in Lodamin-treated group compared with control group (p<0.05). The effect of Lodamin on the regulatory T cell population was examined, showing that Lodamin did not change the number of CD4^+^Foxp3^+^ T cells in lymph nodes (Figure S2), suggesting an increase in number of naive and inactivated T cells (Figure 5C). In order to determine whether the changes in CD4^+^ T cells were mediated by inhibition of MetAP2, its expression in CD4+ cells was analyzed by qPCR. In addition, the expression of P21, previously shown to accumulate during G1 cell cycle arrest in endothelial cells, was also determined. MetAP2 gene expression in CD4^+^ T cells was found to decrease by 55% (p<0.05) in response to Lodamin treatment, while the change in p21 was not statistically significant (Figure 5D).

### Reduced Inflammatory Cytokine Expression in the Eyes of Lodamin Treated Mice

**Figure 6:**
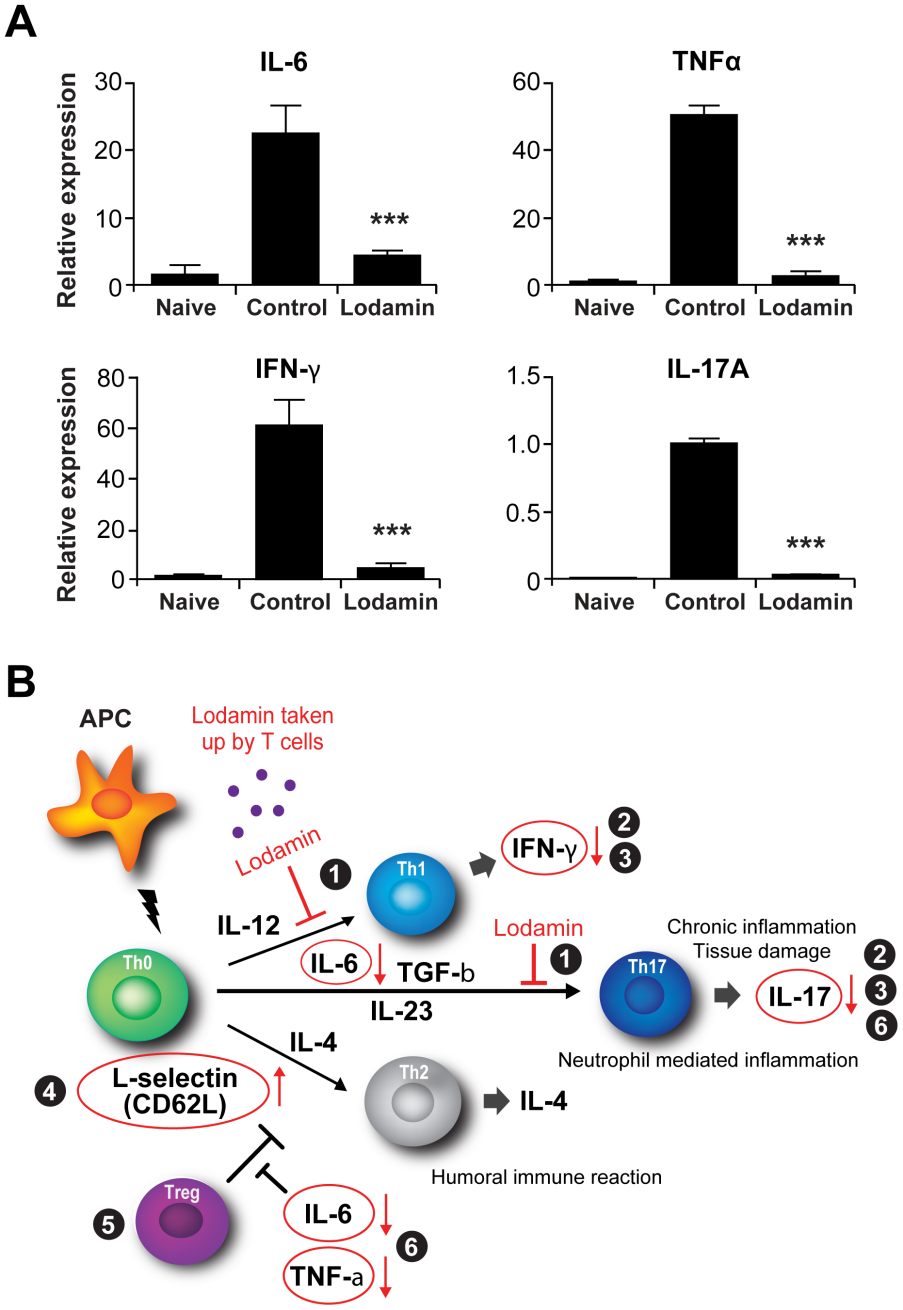
Reduced inflammatory cytokine expression in retina from Lodamin-administered mice, and summary of Lodamin’s activity on T cells. (A) On day 21 after IRBP immunization, retinas were harvested and total RNA was extracted from naive mice (unimmunized control), EAU control group and Lodamin-administered group. The expression of IL-6, TNF, IFN-γ, and IL-17A were analyzed by realtime qPCR (mean ± SEM, n = 5–8, ***p<0.001). (B) Summary of Lodamin’s effects on T cell activity and differentiation *in vitro* and in EAU model (circled, red): Lodamin is taken up by T cells and (1) suppress the activation and differentiation of CD4^+^ T cells to Th1 and Th17. (2) reduces production of IL-17 and IFNγ in splenocytes and activated T cells (3) reduces IFNγ and IL-17 in Lymph T cells from EAU mice (4) increases the expression of homing receptor CD62 lymph node CD4+ T cells (5) do not effect Treg proliferation in lymph nodes (6) reduces the expression of retinal cytokines: IL-6. TNFα, IFNγ, IL-17A.

To investigate the ocular inflammatory response, expression of retinal inflammatory cytokines were examined by qPCR. As shown in Figure 6, major inflammatory cytokines IL-6, TNF, IFN-γ and IL-17A were significantly suppressed in the retinas of Lodamin treated mice compared with control.

## Discussion

Human autoimmune uveitis is regarded as a T cell dependent disease. In the past decade the role of Th1 and Th17 in uveitis and other autoimmune diseases has been established. Therapeutically targeting cellular pathways in these cells is an alternative to broad spectrum steroid treatment [1], [14]. EAU is the standard mouse model that reflects the chronic disease observed in humans. It has been widely used to analyze both the immunopathological mechanisms in uveitis and to develop preventive or therapeutic strategies [15], [16]. EAU can be induced in rodents by immunization with retinal antigens such as interphotoreceptor retinal-binding protein. Using this model, we showed that Lodamin, at a dose optimized based on our previous *in vivo* studies and by dose-response analysis from the corneal micropocket assay (Figure S3), drastically suppressed the overall progression of EAU and ocular inflammation. These clinical effects were associated with altered cell distribution in the lymph nodes, suppression of T cell activation, and diminished antigen-elicited production of IFN-γ and IL-17.

In EAU-bearing mice, histopathological examination clearly showed disorganized retinal architecture including extensive retinal folding with inflammatory cell infiltration and severe photoreceptor damage. In comparison, mice treated with Lodamin (30 mg/kg every other day) had significantly lower infiltration of inflammatory cells and granuloma formation. These dramatic effects were obtained with a dose comparable to the same oral dosage that is antiangiogenic in cancer and ocular neovascularization [10], [17]. The overall effect of Lodamin in EAU is associated with normalization of proinflammatory factors IFN-γ and IL-17 in the lymph node. Moreover, Lodamin prevented the enlargement of lymph nodes seen in EAU control mice, and increased expression of CD62L (L-selectin) in lymph node-originating T cells. CD62L is a homing receptor that mediates entry of naive T cells to peripheral lymph nodes and enables the migration of lymphocytes to lymphoid organs [18]. Thus, down regulation of CD62L is often used as a marker to distinguish naive and antigen-experienced T cells in the blood and lymphoid organs during immune responses. Immune reaction decreases CD62L, triggering effector T lymphocytes to migrate in greater extent to sites of inflammation and non-lymphoid tissues [19]. Following an immediate response to antigen receptor trigger, CD62L from the cell surface of T lymphocytes is cleaved by protease [20], and the transcription of CD62L encoding gene is lost after cytokine-controlled differentiation of effector T cells [21]. Therefore, the increased CD62L expression in Lodamin mice compared to control suggests that Lodamin reduces T cell activation and inhibits the migration of T cells into inflammatory sites. Importantly, the high CD62L expression in Lodamin-administered mice suggests no interference with normal immunity as the levels are comparable with naive state in mice.

MetAP2 is an enzyme that plays a critical role in the regulation of post-translational processing and protein synthesis. MetAP2 inhibition by molecules such as TNP-470 leads to cell cycle arrest in endothelial cells [12], but its effects on T cells, which also express high levels of MetAP2, remained undefined. It has been known for years that antiangiogenic MetAP2 inhibitors from the fumagillin family are effective in suppression of autoimmune collagen-induced arthritis (CIA) in mice and even regress established CIA [22], [23], [24]. Previously, the effects of MetAP2 inhibitors in autoimmune models were largely attributed to antiangiogenic properties since neovascularization plays a central role in these diseases [25]. However, we recently showed that Lodamin, the polymer conjugate of TNP-470, significantly suppressed T cell-mediated delayed-type hypersensitivity (DTH) reaction in mice [10], [17]. In the DTH model, the reduction in angiogenesis was accompanied by decreased vessel permeability as well as fewer infiltrating inflammatory cells. This observation lead us to hypothesize that a MetAP2 inhibitor such as Lodamin may have direct effect on T cells through the MetAP2 cascade. Suppression of the inflammatory response as a primary event could prevent vessel permeability linked to the in situ inflammation. Here we showed that the overall effects of Lodamin in EAU were mediated at least in part by the reduction of MetAP2 expression in purified lymph node CD4^+^ T cells as quantified by qPCR. This can explain the mechanism in which T cell proliferation is suppressed. Interestingly, p21 expression in these cells did not show significant reduction. Accumulation of p21 is associated with G1 cell cycle arrest typical for MetAP2 activity. It is possible that such changes are present at the protein level.

In culture, Lodamin nanoparticles were rapidly taken up by anti-CD3 activated T cells and suppressed their proliferation at 1 nM and 10 nM TNP-470 equivalents, doses comparable to those which inhibit endothelial cell proliferation [10]. *In vitro,* treatment reduced production of the proinflammatory cytokines IFN-γ and IL-17, following Th1 and Th17 polarization, respectively (see scheme Figure 6B). However, no alteration of the T-bet (Th1 specific transcription factor) and RORγt, (Th17 transcriptional factor) expression were observed between control and Lodamin-administered mice (data not shown). Moreover, flow cytometric analysis of Foxp3 in CD4+ cells revealed that Lodamin had no effect on T regulatory cell population (Figure S2). When taken together, these results suggest that when naive T cells proliferate from exposure to an antigen-elicited dendritic cell trigger, the presence of Lodamin inhibits T cell proliferation and differentiation into Th1 and Th17 cells. This results in reduced production of IFN-γ by Th1 cells and of IL-17 by Th17 cells without affecting other T regulatory cell populations.

As previously shown, Lodamin’s novel formulation improves TNP-470’s safety profile in mice and does not cause general systemic side effects such as weight loss or behavioral changes at the therapeutic dose [10]. Moreover, unlike TNP-470, Lodamin is orally available, making it advantageous for acute and chronic treatments in uveitis as well as other autoimmune disease. Future studies aim to address Lodamin’s ability to regress established disease phenotypes. Since Lodamin’s mechanism of action is different than that of the current treatments of immunosuppressive agents and biologics, it can potentially have synergistic effects in combination with these treatments. Thus combination therapy may allow the reduction of the dose and subsequent side effects of the existing immunosuppressive drugs.

## Materials and Methods

### Mice

Female C57BL/6J (H-2^b^) mice were purchased from Jackson Laboratories (Bar Harbor, ME). All mice used were between 6 and 10 weeks of age and maintained in specific pathogen-free conditions in accordance with institutional guidelines. All protocols were approved by the Institutional Animal Care and Use Committee at Boston Children’s Hospital and all experiments conformed to the Association for Research in Vision and Ophthalmology Statement for the Use of Animals in Ophthalmic and Vision Research (Protocol No. A09-07-1455 BCH).

### Antibodies

All antibodies were purchased from eBioscience. The following specific antibodies for activating and staining T cells were used: purified hamster anti-mouse CD3e antibody (clone 145-2C11), anti-mouse FcR-allophycocyanin (APC), monoclonal rat anti-mouse CD4 (RM4-5), phycoerythrin (PE)-conjugated anti-mouse CD62L (MEL-14), fluorescence isothiocyanate (FITC)-conjugated rat anti-mouse IFN-γ (XMG1.2), PE-conjugated, monoclonal rat anti-mouse IL-17 (eBio17B7), FITC-conjugated rat anti-mouse CD4(RM4-5), PE-conjugated anti-CD25(PC61.5) and APC-conjugated anti-Foxp3(FJK16-s).

### Preparation of Lodamin and Fluorescently Labeled Micelles

Lodamin was prepared as previously described [10]. Briefly, TNP-470 was conjugated to a diblock co-polymer methoxypolyethylene glycol-polylactic acid (mPEG-PLA) using a two-step reaction. In the first step, succinated mPEG_2000_-PLA_1000_ (Advanced Polymers Materials) was reacted with ethylenediamine (Sigma-Aldrich) using ethyl (diethylaminopropyl) carbodiimide (EDC) and a catalyst N-hydroxysuccinimide (NHS). In the second step, the amine-containing polymer was reacted with TNP-470 and dialyzed to form nanomicelles. The micelles were then lyophilized and stored at −20°C in a dry environment until use. For fluorescently labeled Lodamin, a commonly used hydrophobic marker 6-coumarin (Sigma-Aldrich) at 0.1% wt/wt was added to the polymeric solution before the final dialysis step. The doses of Lodamin as noted are presented as TNP-470 equivalent.

### Cell Culture

RPMI 1640 medium (GIBCO) supplemented with 10% fetal calf serum (FCS) and 100U/ml penicillin-streptomycin was used for all cell cultures. Whole splenocytes were activated with immobilized anti-CD3 monoclonal antibody (mAb) (2C11; 1 µg/ml). For preparation of naive CD4^+^ cells, cells were sorted by negative selection using magnetic beads plus anti-CD8α, anti-B220, anti-Mac-1, and anti-NK1.1 Abs (eBioscience). Purified CD4^+^ cells were then positively selected using anti-CD62 ligand (CD62L) Ab (CD4^+^CD62L^+^ cells >95%). Recall responses of secondary lymphoid tissue were measured 21 days after immunization with IRBP_1–20_ peptide (0–100 µmol/l).

### T Cell Uptake Study

Purified CD4^+^ cells were pre-activated with anti-CD3 Ab (1 µg/ml) for 24 h and 10 mg/ml of 6-coumarin labeled Lodamin was added and incubated for specified time points (5, 10, 30 min). After incubation, cells were washed twice with PBS and subjected to flow cytometry. The fluorescently labeled cells were detected by FL-1^high^ population of live cells.

### Leukocyte Proliferation Assay using CFSE Labeling

In order to evaluate the effect of Lodamin on leukocyte proliferation we used CFSE-labeling. For this assay whole splenocytes were labeled with 1 µM CFSE (Fluka, Buchs, Switzerland) in serum-free HBSS for 8 min at room temperature. The labeling was then halted by adding excess volume of HBSS containing FCS. The labeled cells were washed twice in complete medium and then seeded and stimulated as described in the text. Splenocytes (1×10^5^/well in round-bottom 96 well plates) were stimulated with anti-CD3 mAb at a final concentration of 1 µg/ml. After 72 h, surface CD4^+^ antigen was stained with allophycocyanin (APC)-conjugated anti-CD4 and CFSE dilution was analyzed by flow cytometry.

### EAU Induction and Evaluation

Wild type (WT) female C57BL/6J mice were immunized with human IRBP peptide 1–20 (GPTHLFQPSLVLDMAKVLLD), as previously described [26]. The mice were immunized subcutaneously (s.c.) in one footpad and the top of the head with the peptide in 0.2 ml emulsion of ***Complete Freund's Adjuvant*** (CFA) in 1∶1, vol/vol, supplemented with mycobacterium tuberculosis strain H37RA to 2.5 mg/ml, and then inoculated intraperitoneally (i.p.) with 100 ng of pertussis toxin. Lodamin was administered orally (30 mg/kg) every other day and ophthalmic examinations were carried out following immunization. Tropicamide (0.5%) was applied to the eyes to induce mydriasis, and the fundus was examined using a slitlamp microscope. EAU severity was assessed both clinically and histopathologically in a blinded manner as described by Thurau et al [27].

### Evaluation of IRBP-specific Cytokine Production in Lymphatic CD4^+^ T Cells

On day 21 post-immunization, cervical, submandibular and inguinal lymph nodes were harvested, and single cell suspensions were prepared with a culture medium (RPMI1640 medium, Sigma) supplemented with 10% fetal bovine serum (FBS, Invitrogen) and penicillin/streptomycin (Gibco). CD4^+^ enriched cells were prepared using anti-CD4 Microbeads (#130-1-049-201; Miltenyi Biotec, Gladbach, Germany) and MS columns (#130-042-201; Miltenyi) with a MiniMACS™ separator (#130-090-312; Miltenyi Biotec). The suspended CD4^+^ T cells (2×10^5^cells/200 µl/well) were incubated with 50 µg/ml of mitomycin-C (MMC)-treated splenocytes as antigen presenting cells (APC) (1×10^6^ cells/200 µl/well) and indicated concentrations of IRBP peptide in a 96-well flat-bottom microtiter plate for 48 h at 37°C. IFN-γ and IL-17 levels in culture supernatants were measured by an enzyme-linked immunosorbent assay (ELISA) according to the manufacturer’s directions (eBioscience).

### Flow Cytometric Analysis

Lymph node cells were prepared as described above and stained with APC anti-CD4 and PE anti-CD62L mAb for detection of effector T cells. Cells were washed with PBS, resuspended, stained with propidium iodide (1 µg/ml; Invitrogen) and live cells were analyzed by flow cytometry using propidium iodide-negative gate. To stain cells for their cytokine content, cells were restimulated with 50 ng/ml phorbol 12-myristate 13-acetate and 1 µg/ml ionomycin (Sigma-Aldrich) in the presence of Brefeldin A (eBioscience) for 5 h, stained with anti-CD4 antibody, fixed, and permeabilized using intracellular fixation buffer and permeabilization buffer (eBioscience) followed by FITC anti-IFN-γ and PE anti-IL-17 staining. For the analysis of Foxp3^+^T_reg_ cells, single-cell suspensions prepared from the lymph nodes were stained with fluorophore-conjugated monoclonal antibodies: FITC anti-CD4, PE anti-CD25 (PC61.5, eBioscience), and APC anti-Foxp3. Expression of the surface markers and cytokines was analyzed using FACS Calibur flow cytometer (BD Biosciences, Franklin Lakes, NJ) and FlowJo software (Treestar, Ashland, OR).

### Quantitative TaqMan RT-PCR

Total RNA extracted from the retina using TRIzol reagent (Invitrogen) was used as a template for cDNA synthesis. cDNA was obtained using the AffinityScript qPCR cDNA synthesis kit (Stratagene, La Jolla, CA) according to the manufacturer’s instructions. Quantitative real-time PCR (qPCR) was performed using the MX3005P device (Stratagene, La Jolla, CA). cDNA was amplified in a 10 µg/ml final reaction mix containing TaqMan Universal PCR Master Mix (Applied Biosystems, Foster City, CA) and corresponding TaqMan Gene Expression Assays (Mm99999064_m1 (IL-6), Mm99999068_m1 (TNF), Mm00439619_m1 (IL-17A), Mm99999071_m1 (IFN-γ) and Mm00607939_s1 (β-actin, Applied Biosystems). Signals were analyzed by the MxPro QPCR software version 3.0 (Stratagene). The comparative threshold cycles (Ct) method for relative quantification was used, whereby all Ct are first normalized to the expression of an endogenous control (GAPDH). The normalized values were then divided by the average delta Ct value of samples from unimmunized control. The expression was represented as a fold change relative to retinas from unimmunized mice.

### Statistical Analysis

Statistical analysis was performed using Prism v5.0a (Graphpad Software). Data are shown as mean ± standard error of the mean (SEM). Unpaired Student’s t-test for parametric data (cytokine production) and Mann-Whitney U-test for non-parametric data (EAU scores, evaluation of cell activation markers and Foxp3 expression, and retinal qRT-PCR) were used to analyze differences between groups of mice. P values less than 0.05 were considered significant.

## Supporting Information

Figure S1 **Relative MetAP2 gene expression in different human normal primary tissues and cells.** Graph show the top 20 tissues and cells with the highest expression of MetAp2 gene. CD4+ thymic cells have high expression of MetAp2 gene compared with other tissues, except of endothelial cells and hematopoietic bone marrow stem cells. The graph represents an analysis of database that contains expression profiles for ∼12,000 genes with NCBI GeneID entry across 126 primary human tissues. The enrichment score is comparable between genes, thus allowing ranking of genes in each tissue profiled. The software was described previously [28] and is available at: http://xavierlab2.mgh.harvard.edu/EnrichmentProfiler/index.html.
(PDF)

Figure S2 **Lodamin does not change the CD4+Foxp3+ T regulatory cell population in lymph nodes. intracellular staining for Foxp3 expression and mean fluorescence intensity (MFI) (** ***n = 5*** **).**
(PDF)

Figure S3 **Lodamin dose-response effect in The Cornea Micropocket Assay.** To evaluate the maximal ophthalmic effect of oral administered Lodamin, a short corneal micropocket angiogenesis assay was performed as previously detailed (see ref. Pellets containing 80 ng carrier-free recombinant human bFGF or 160 ng (R&D Systems) were implanted into micropockets created in the cornea of anesthetized mice. Mice were treated daily with either 15 mg/kg, 30 mg/kg or 60 mg/kg TNP-470 equivalent of Lodamin for 4 d, and then the vascular growth area was measured using a slit lamp. The area of neovascularization was calculated as vessel area by the product of vessel length measured from the limbus and clock hours around the cornea, using the following equation: vessel area (mm2) = (πx clock hours×vessel length (mm)×0.2 mm). (n = 10, mean ± s.d).
(TIF)

## References

[1] Horai R, Caspi RR (2011) Cytokines in autoimmune uveitis. J Interferon Cytokine Res 31: 733–744. 21787221 10.1089/jir.2011.0042 PMC3189550

[2] de Smet MD, Taylor SR, Bodaghi B, Miserocchi E, Murray PI, et al (2011) Understanding uveitis: the impact of research on visual outcomes. Prog Retin Eye Res 30: 452–470. 21807112 10.1016/j.preteyeres.2011.06.005

[3] Bettelli E, Oukka M, Kuchroo VK (2007) T(H)-17 cells in the circle of immunity and autoimmunity. Nat Immunol 8: 345–350. 17375096 10.1038/ni0407-345

[4] Amadi-Obi A, Yu CR, Liu X, Mahdi RM, Clarke GL, et al (2007) TH17 cells contribute to uveitis and scleritis and are expanded by IL-2 and inhibited by IL-27/STAT1. Nat Med 13: 711–718. 17496900 10.1038/nm1585

[5] Chi W, Zhu X, Yang P, Liu X, Lin X, et al (2008) Upregulated IL-23 and IL-17 in Behcet patients with active uveitis. Invest Ophthalmol Vis Sci 49: 3058–3064. 18579762 10.1167/iovs.07-1390

[6] McEwen BS, Biron CA, Brunson KW, Bulloch K, Chambers WH, et al (1997) The role of adrenocorticoids as modulators of immune function in health and disease: neural, endocrine and immune interactions. Brain Res Brain Res Rev 23: 79–133. 9063588 10.1016/s0165-0173(96)00012-4

[7] Smolen JS, Emery P (2011) Infliximab: 12 years of experience. Arthritis Res Ther 13 Suppl 1S2. 21624181 10.1186/1478-6354-13-S1-S2 PMC3123963

[8] Ogata A, Tanaka T (2012) Tocilizumab for the treatment of rheumatoid arthritis and other systemic autoimmune diseases: current perspectives and future directions. Int J Rheumatol 2012: 946048. 22315615 10.1155/2012/946048 PMC3270395

[9] Hansel TT, Kropshofer H, Singer T, Mitchell JA, George AJ (2010) The safety and side effects of monoclonal antibodies. Nat Rev Drug Discov 9: 325–338. 20305665 10.1038/nrd3003

[10] Benny O, Fainaru O, Adini A, Cassiola F, Bazinet L, et al (2008) An orally delivered small-molecule formulation with antiangiogenic and anticancer activity. Nat Biotechnol 26: 799–807. 18587385 10.1038/nbt1415 PMC2803109

[11] Griffith EC, Su Z, Niwayama S, Ramsay CA, Chang YH, et al (1998) Molecular recognition of angiogenesis inhibitors fumagillin and ovalicin by methionine aminopeptidase 2. Proc Natl Acad Sci U S A 95: 15183–15188. 9860943 10.1073/pnas.95.26.15183 PMC28017

[12] Zhang Y, Griffith E, Sage J, Jacks T, Liu J (2000) Cell cycle inhibition by the anti-angiogenic agent TNP-470 is mediated by p53 and p21WAF1/CIP1. Proc Natl Acad Sci U S A 97: 6427–6432. 10841547 10.1073/pnas.97.12.6427 PMC18619

[13] Mauriz J, Gonzalez P, Duran M, Molpeceres V, Culebras J, et al (2007) Cell-cycle inhibition by TNP-470 in an in vivo model of hepatocarcinoma is mediated by a p53 and p21WAF1/CIP1 mechanism. Transl Res 149: 46–53. 17196522 10.1016/j.trsl.2006.07.004

[14] Damsker JM, Hansen AM, Caspi RR (2010) Th1 and Th17 cells: adversaries and collaborators. Ann N Y Acad Sci 1183: 211–221. 20146717 10.1111/j.1749-6632.2009.05133.x PMC2914500

[15] Caspi RR (2003) Experimental autoimmune uveoretinitis in the rat and mouse. Curr Protoc Immunol Chapter 15: Unit 15 16. 10.1002/0471142735.im1506s53 18432901

[16] Caspi RR, Silver PB, Luger D, Tang J, Cortes LM, et al (2008) Mouse models of experimental autoimmune uveitis. Ophthalmic Res 40: 169–174. 18421234 10.1159/000119871 PMC2735820

[17] Benny O, Nakai K, Yoshimura T, Bazinet L, Akula JD, et al.. (2010) Broad spectrum antiangiogenic treatment for ocular neovascular diseases. PLoS One 5. 10.1371/journal.pone.0012515 PMC2931703 20824139

[18] Weninger W, Carlsen HS, Goodarzi M, Moazed F, Crowley MA, et al (2003) Naive T cell recruitment to nonlymphoid tissues: a role for endothelium-expressed CC chemokine ligand 21 in autoimmune disease and lymphoid neogenesis. J Immunol 170: 4638–4648. 12707342 10.4049/jimmunol.170.9.4638

[19] Mora JR, von Andrian UH (2006) T-cell homing specificity and plasticity: new concepts and future challenges. Trends Immunol 27: 235–243. 16580261 10.1016/j.it.2006.03.007

[20] Venturi GM, Tu L, Kadono T, Khan AI, Fujimoto Y, et al (2003) Leukocyte migration is regulated by L-selectin endoproteolytic release. Immunity 19: 713–724. 14614858 10.1016/s1074-7613(03)00295-4

[21] Chao CC, Jensen R, Dailey MO (1997) Mechanisms of L-selectin regulation by activated T cells. J Immunol 159: 1686–1694. 9257829

[22] Peacock DJ, Banquerigo ML, Brahn E (1992) Angiogenesis inhibition suppresses collagen arthritis. J Exp Med 175: 1135–1138. 1372645 10.1084/jem.175.4.1135 PMC2119184

[23] Oliver SJ, Banquerigo ML, Brahn E (1994) Suppression of collagen-induced arthritis using an angiogenesis inhibitor, AGM-1470, and a microtubule stabilizer, taxol. Cell Immunol 157: 291–299. 7518753 10.1006/cimm.1994.1223

[24] Brahn E, Schoettler N, Lee S, Banquerigo ML (2009) Involution of collagen-induced arthritis with an angiogenesis inhibitor, PPI-2458. J Pharmacol Exp Ther 329: 615–624. 19218530 10.1124/jpet.108.148478 PMC2672877

[25] Clavel G, Bessis N, Boissier MC (2003) Recent data on the role for angiogenesis in rheumatoid arthritis. Joint Bone Spine 70: 321–326. 14563458 10.1016/s1297-319x(03)00088-5

[26] Avichezer D, Silver PB, Chan CC, Wiggert B, Caspi RR (2000) Identification of a new epitope of human IRBP that induces autoimmune uveoretinitis in mice of the H-2b haplotype. Invest Ophthalmol Vis Sci 41: 127–131. 10634611

[27] Thurau SR, Chan CC, Nussenblatt RB, Caspi RR (1997) Oral tolerance in a murine model of relapsing experimental autoimmune uveoretinitis (EAU): induction of protective tolerance in primed animals. Clin Exp Immunol 109: 370–376. 9276535 10.1046/j.1365-2249.1997.4571356.x PMC1904752

[28] Benita Y, Cao Z, Giallourakis C, Li C, Gardet A, et al (2010) Gene enrichment profiles reveal T-cell development, differentiation, and lineage-specific transcription factors including ZBTB25 as a novel NF-AT repressor. Blood 115: 5376–5384. 20410506 10.1182/blood-2010-01-263855 PMC2902135

