# Supplementary figures and images for: Suppression of Autoimmune Retinal Inflammation by an Antiangiogenic Drug
Source: PLoS One. 2013 Jun 13;8(6):e66219. doi: 10.1371/journal.pone.0066219 (PMC3681944; doi:10.1371/journal.pone.0066219)

# Expression of MetAP2

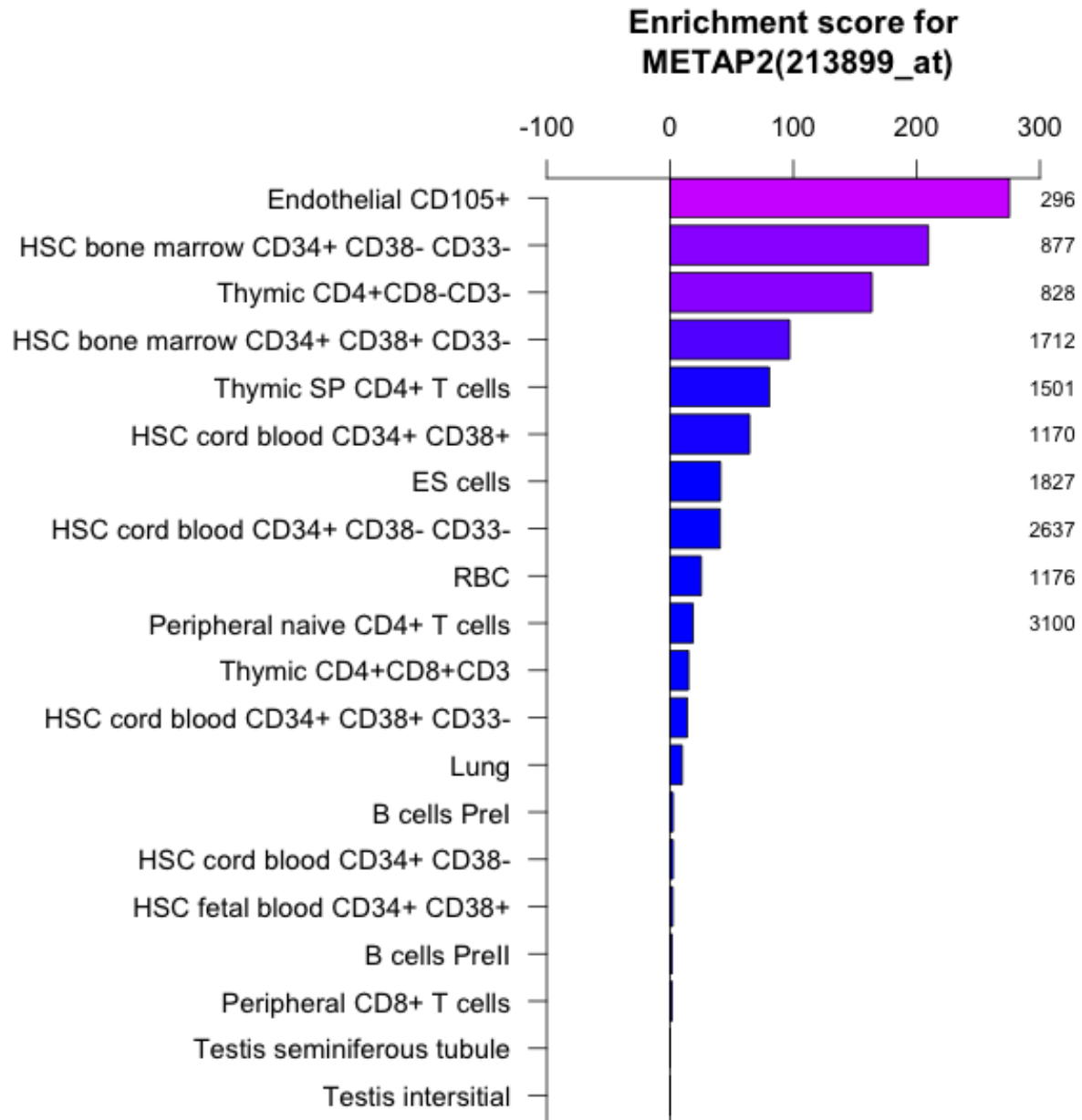

Supplement: Figure S1 — Relative MetAP2 gene expression in different human normal primary tissues and cells. Graph show the top 20 tissues and cells with the highest expression of MetAp2 gene. CD4+ thymic cells have high expression of MetAp2 gene compared with other tissues, except of endothelial cells and hematopoietic bone marrow stem cells. The graph represents an analysis of database that contains expression profiles for ∼12,000 genes with NCBI GeneID entry across 126 primary human tissues. The enrichment score is comparable between genes, thus allowing ranking of genes in each tissue profiled. The software was described previously [28] and is available at: http://xavierlab2.mgh.harvard.edu/EnrichmentProfiler/index.html. (PDF) [file pone.0066219.s001.pdf]

## CD4<sup>+</sup>Foxp3<sup>+</sup>

control-1...CD4-positive

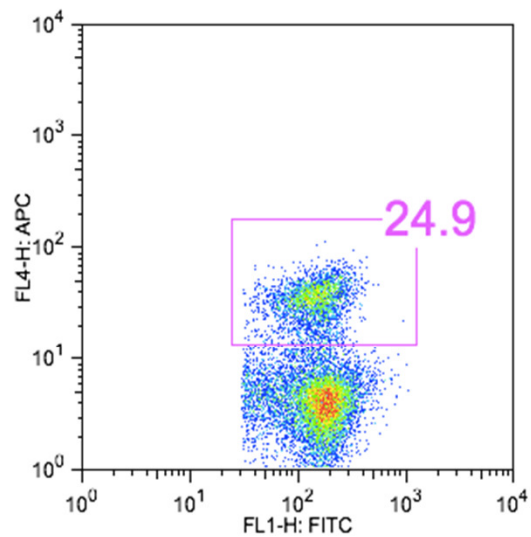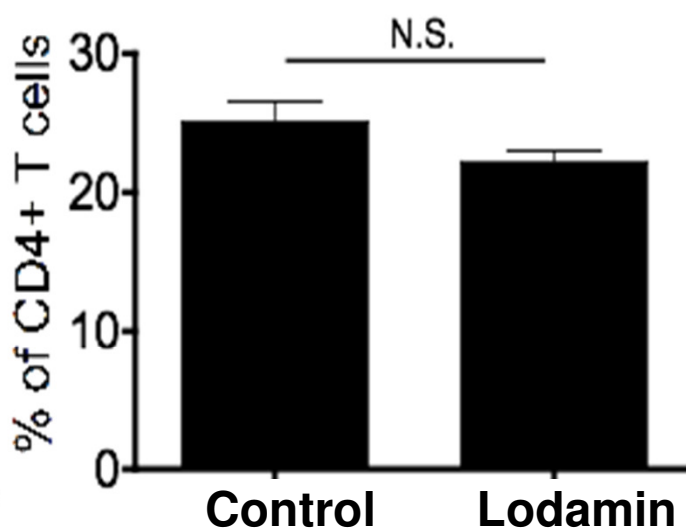

Lodamin-4...CD4-positive

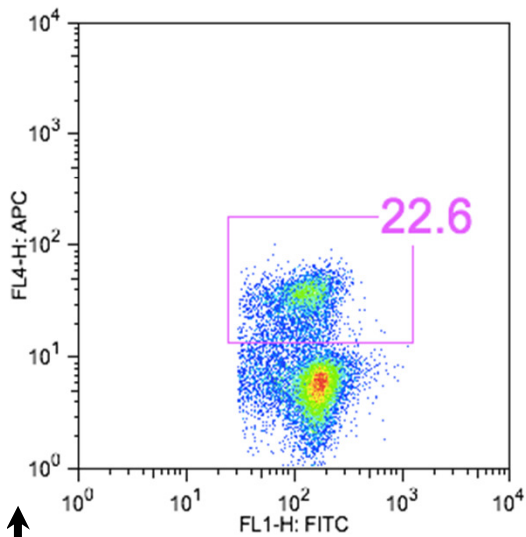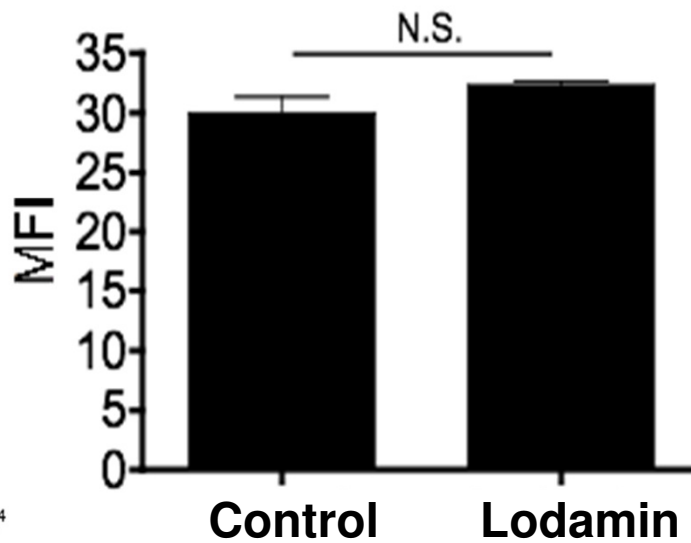

Foxp3  
CD4

Supplement: Figure S2 — Lodamin does not change the CD4+Foxp3+ T regulatory cell population in lymph nodes. intracellular staining for Foxp3 expression and mean fluorescence intensity (MFI) ( n = 5 ). (PDF) [file pone.0066219.s002.pdf]

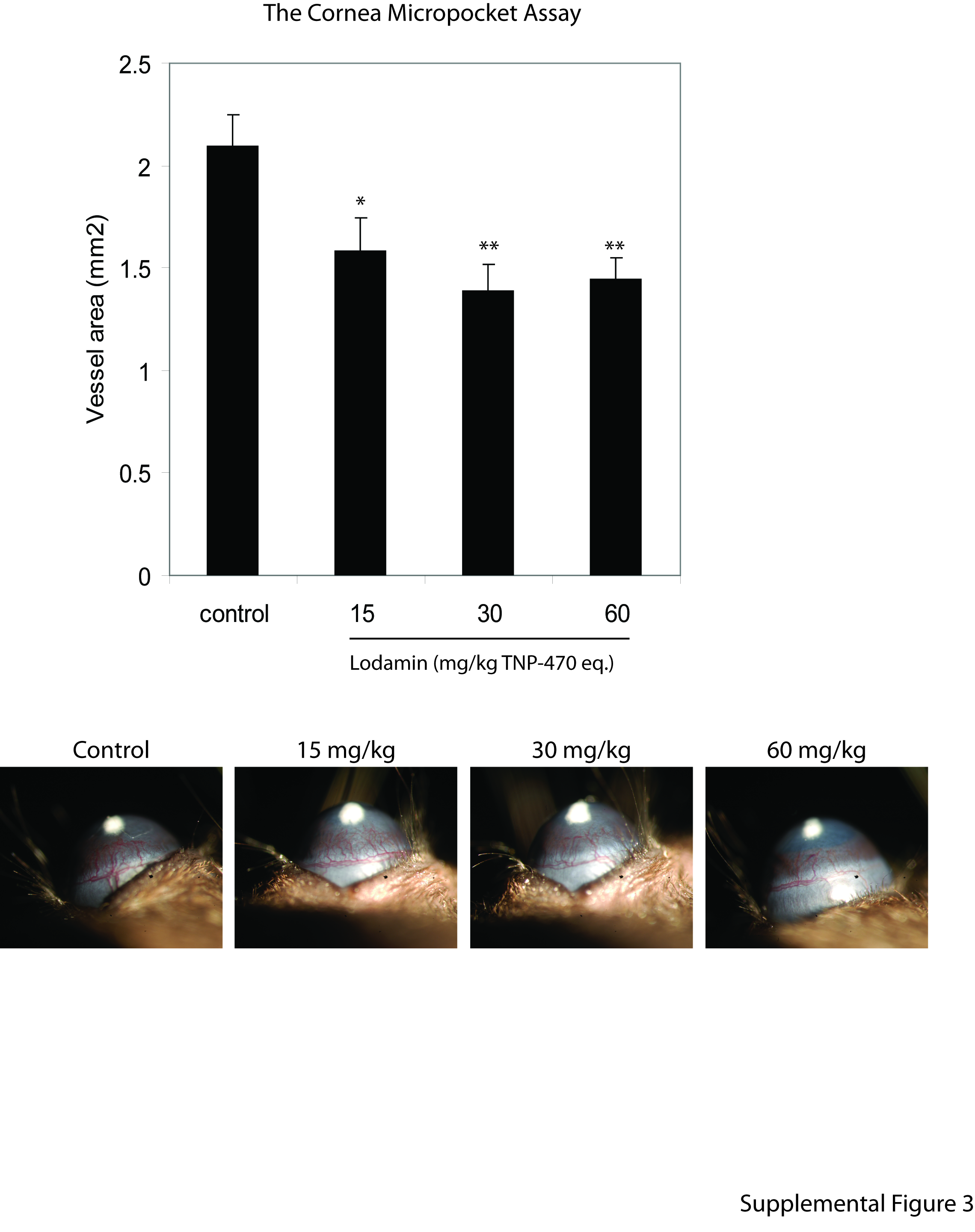

Supplement: Figure S3 — Lodamin dose-response effect in The Cornea Micropocket Assay. To evaluate the maximal ophthalmic effect of oral administered Lodamin, a short corneal micropocket angiogenesis assay was performed as previously detailed (see ref. Pellets containing 80 ng carrier-free recombinant human bFGF or 160 ng (R&D Systems) were implanted into micropockets created in the cornea of anesthetized mice. Mice were treated daily with either 15 mg/kg, 30 mg/kg or 60 mg/kg TNP-470 equivalent of Lodamin for 4 d, and then the vascular growth area was measured using a slit lamp. The area of neovascularization was calculated as vessel area by the product of vessel length measured from the limbus and clock hours around the cornea, using the following equation: vessel area (mm2) = (πx clock hours×vessel length (mm)×0.2 mm). (n = 10, mean ± s.d). (TIF) [file pone.0066219.s003.tif]
